# Relating latent factors of reasoning, affect, and cognition to the delusional experience

**DOI:** 10.1038/s41537-026-00750-1

**Published:** 2026-04-24

**Authors:** Annalise Halverson, Andrew R. Kittleson, Jinyuan Liu, Suzanne HW So, Julia M. Sheffield

**Affiliations:** 1 Department of Psychiatry and Behavioral Sciences, Vanderbilt University Medical Center, Nashville, TN USA; 2 Department of Biostatistics, Vanderbilt University Medical Center, Nashville, TN USA; 3 Department of Psychology, The Chinese University of Hong Kong, Hong Kong, China

**Keywords:** Human behaviour, Psychology

## Abstract

Reasoning biases play a crucial role in the formation and maintenance of delusions. In psychosis, it is unclear whether these biases cluster onto similar underlying factors or represent independent constructs. The current study presents data on task-based reasoning biases and self-reported cognitive biases, as well as cognition and affect. Using these data, we aimed to understand mechanisms underlying facets of clinical delusional severity and broader unusual thought content. Participants with schizophrenia-spectrum disorders (SSD; *N* = 75) were enrolled following hospitalization for an acute psychotic episode. Non-clinical comparison participants (NCC; *N* = 70) were recruited for comparison of affect, cognition, and reasoning biases. Exploratory factor analysis and multivariable linear regressions were performed in the SSD group only. Core clinical delusions and broader unusual thought content, alongside preoccupation, conviction, and distress were used as outcome measures. SSD participants had more severe reasoning biases, cognitive deficits, and negative affect than NCC. In SSD, factor analysis revealed four latent constructs representing belief updating, cognitive biases, affective disturbances, and general cognitive ability. Affect was significantly associated with delusional preoccupation and distress. Belief updating was the primary factor associated with conviction. Cognitive biases related to the number of unusual beliefs endorsed, but no other aspects of severity. General cognitive ability was unrelated to all facets of delusional severity in our sample. Affective disturbances and belief updating map onto distinct aspects of clinical delusions in SSD; delusion severity is not robustly related to cognitive impairment. Reducing delusional conviction may require treatments focused on how new evidence is integrated into existing beliefs.

## Background

Delusions are a core feature of psychosis, reflecting erroneous and enduring beliefs that arise from unreliable interpretations of external reality^1^. While medication and therapy can improve delusion severity, these experiences remain difficult to treat^2^. Advancements in psychotherapy and cognitive neuroscience continue to seek a precise understanding of the mechanisms contributing to delusional thinking^3^.

Some of the earliest mechanistic models of delusions implicated reasoning biases—systematic distortions of judgment—in the development and maintenance of unusual beliefs^4^. Reasoning biases are prominent in psychosis, with one study showing 50% prevalence at first assessment^5^. If beliefs are based on reasoned inferences about the world, then perhaps delusional beliefs reflect a fallacy in this process. Several biases have been identified as relevant to delusions. Jumping to conclusions (JTC) reflects the tendency to make hasty decisions using little data. JTC is a well-studied bias that is unique to delusional psychosis^6,7^, and delineates an overarching assignment of salience toward incoming information^8,9^. The bias against disconfirmatory evidence (BADE) captures a failure to update beliefs in the face of conflicting evidence^10^. BADE may also be related to paranoid thinking^11^ and serve as a promising intermediary target in interventions for delusions^12,13^. As such, JTC and BADE can be understood through the broader framework of Bayesian belief updating, originally theorized by Hemsley and Garety^14^ to reflect a deviance from optimal integration of incoming information.

Additionally, other cognitive biases, such as external attribution, selective attention to threat, and belief inflexibility, can reflect self-perceptions of biases. These perceptions may influence the way information in the environment is processed (e.g., attention to threat) as well as inferences about their source and meaning (e.g., external attribution). Similarly, poor cognitive insight—awareness into one’s own judgment and reasoning processes—may exacerbate reasoning biases by increasing self-certainty and reducing self-reflection^15^. Though “reasoning biases” or “cognitive biases” are often referenced as a whole, these behaviorally derived and self-reported biases may reflect distinct latent constructs.

In addition, we must keep in mind that these processes exist against the backdrop of well-studied neurocognitive impairments seen in schizophrenia-spectrum disorders (SSD^16^). Cognitive ability impacts behavior on assessments of these biases, leading some to worry that these measures reflect cognitive impairment rather than a sound bias in information processing. To this point, prior work has suggested that biases such as JTC may simply reflect cognitive deficits in SSDs^17^. Equivocal findings leave us to wonder whether cognitive biases are best characterized as another dimension of neurocognitive impairment or if they should be considered distinct^11^.

Beyond cognitive and reasoning biases, increasing evidence suggests a core role of affective processes in delusion severity. Severe delusional presentations are often linked with higher affective disturbances^18^ and certain processes—including anxiety and depression—are consistently associated with severity of delusions^19,20^. Further, treatment of affective processes improves delusion severity^21,22^. A deeper understanding of the relationship between affective disturbances, including anxiety and depression, and different facets of delusions remains critical for optimizing treatments.

Finally, delusional thinking is multidimensional^23^. Factor analyses reveal three characteristics: conviction (i.e., how strongly held the belief is), preoccupation (i.e., how much time is spent thinking about it), and distress (i.e., the degree of negative feelings experienced alongside it)^1^. Preoccupation and conviction are more resistant to antipsychotic treatment and warrant continued therapeutic attention^24^. Distinct causal factors may influence specific facets of delusion severity, again guiding expectations for treatment response. Delusional thinking exists on a spectrum, ranging from a specific crystallized delusion to a broader range of unusual thought content endorsed by the individual (delusional ideation). Both types of delusional thinking can be characterized by conviction, preoccupation, and distress, but they have demonstrated different relationships with reasoning/cognitive biases, affect, and cognitive deficits^25^.

While prior work has examined the relationships among contributory factors and delusion severity in isolation, few have investigated these variables together in a well-characterized cohort of individuals with SSDs. The current study aims to (1) test the underlying factor structure of task-based JTC and BADE, self-reported cognitive biases and affective symptoms, and a standardized assessment of neurocognitive ability in SSD; and (2) determine how these different factors relate to facets of severity for both core clinical delusions and delusional ideation more broadly. To our knowledge, this is the first study to simultaneously examine task-based belief updating indices, self-reported cognitive biases, affect, and neurocognition in one factor-analytic model, and further link these components to multidimensional measures of delusion severity in a well-characterized SSD sample. The goal of the study is to understand whether these contributory factors are in fact distinct, thereby representing different targets for intervention.

## Results

Demographics, including delusional severity/ideation, can be seen in Table 1. On average, SSD participants showed moderate delusional severity on the PSYRATS and significantly higher delusional endorsement and severity on the PDI, compared to NCC.

**Table 1:** Demographics summary of participants.

	NCC, *N* = 70^a^	SSD, *N* = 75^a^	*p*-value^b,c^
Age			0.7
Mean (SD)	29.3 (7.6)	28.9 (8.1)	
Median	27.0	27.0	
Min, Max	19.0, 53.0	18.0, 54.0	
Gender			0.9
Man	48 (69%)	48 (65%)	
Woman	21 (30%)	25 (34%)	
Non-binary	1 (1.4%)	1 (1.4%)	
Ethnicity (not Hispanic)	62 (89%)	69 (92%)	0.7
Race			0.5
Asian	4 (5.8%)	2 (2.7%)	
Black	19 (28%)	29 (39%)	
Middle Eastern	1 (1.4%)	1 (1.3%)	
Multiracial	0 (0%)	2 (2.7%)	
Other	2 (2.9%)	2 (2.7%)	
White	43 (62%)	39 (52%)	
Years of education			<0.001***
Mean (SD)	17.3 (2.5)	14.0 (2.1)	
Median	16.0	14.0	
Min, Max	12.0, 25.0	8.0, 21.0	
Parental education			0.4
Mean (SD)	15.4 (2.8)	15.0 (2.5)	
Median	16.0	15.0	
Min, Max	6.0, 24.0	9.5, 22.0	
Premorbid IQ (WTAR)			<0.001***
Mean (SD)	114.7 (9.9)	104.5 (12.7)	
Median	117.0	107.0	
Min, Max	73.0, 126.0	80.0, 127.0	
Delusional severity (PSYRATS)			
Mean (SD)		11.4 (6.6)	
Median		13	
Min, Max		0.0, 23.0	
Delusional endorsement (PDI)			<0.001***
Mean (SD)	1.7 (2.3)	7.4 (5.0)	
Median	0.0	7.0	
Min, Max	0.0, 10.0	0.0, 20.0	
Delusional severity (PDI)			<0.001***
Mean (SD)	6.7 (1.8)	8.7 (2.6)	
Median	7.0	8.6	
Min, Max	3.5, 11.0	3.2, 15.3	

^a^n (%).

^b^Welch two Sample t-test; Pearson’s Chi-squared test.

^c^***p < 0.001.

### Group differences

Controlling for age and sex, we found significant differences between our SSD and NCC groups on all variables. All group differences reflected greater impairment and/or more symptomatology in SSD, apart from BCIS, which revealed better self-reported cognitive insight in SSD (Table 2).

**Table 2:** Group differences between SSD (*N* = 75) and NCC (*N* = 70) on variables of interest.

Measure	F-statistic	${\eta }_{p}^{2}[{\rm{confidence\; interval}}]$	*p*-value
Immediate verbal learning (SCIP)	F(1,141) = 58.48	0.29 [0.19, 1.00]	<0.001
Delayed verbal learning (SCIP)	F(1,141) = 58.32	0.29 [0.19, 1.00]	<0.001
Working memory (SCIP)	F(1,141) = 44.93	0.24 [0.15, 1.00]	<0.001
Verbal fluency (SCIP)	F(1,141) = 18.84	0.12 [0.05, 1.00]	<0.001
Cognitive insight (BCIS)	F(1,141) = 5.64	0.04 [0.00, 1.00]	0.019
Depression (BDI-II)	F(1,141) = 53.70	0.28 [0.18, 1.00]	<0.001
Perseverative thinking (PTQ)	F(1,141) = 45.69	0.24 [0.15, 1.00]	<0.001
Anxiety (BAI)	F(1,141) = 52.19	0.27 [0.17, 1.00]	<0.001
Evidence integration impairment (BADE)	F(1,141) = 11.06	0.07 [0.02, 1.00]	0.001
Positive response bias (BADE)	F(1,141) = 6.52	0.04 [0.01, 1.00]	0.012
Draws to decision (JTC)	F(1,141) = 11.15	0.07 [0.02, 1.00]	0.001
Switching behavior (JTC)	F(1,141) = 19.91	0.12 [0.05, 1.00]	<0.001
Decision threshold (JTC)	F(1,141) = 5.78	0.04 [0.00, 1.00]	0.018
Cognitive biases (DACOBS)	F(1,141) = 63.79	0.31 [0.21, 1.00]	<0.001

All ANCOVAS controlled for age and sex. Partial eta-squared (${\eta }_{p}^{2}$) as well as confidence intervals are reported as effect sizes alongside *p*-values.

### Factor analysis

Bartlett’s (*χ*^2^(210) = 860.061, *p* < 0.001) and KMO tests (MSA = 0.709) indicated moderate correlation and sampling adequacy, and parallel analysis revealed four underlying factors. A graph of the parallel analysis can be found in the Supplementary Fig. S1.

The four-factor solution explained 50% of total variance in the dataset. Factors were renamed based on their contents: Affect, Belief Updating, Cognitive Biases, and General Cognitive Ability. The internal reliability of each factor was further tested alongside fit indices. We found the reliability was good for Cognitive Biases (0.864) and Affect (0.81), and acceptable for Belief Updating (0.759) and General Cognitive Ability (0.629). The root mean square of the residuals (RMSR) indicated overall good fit (0.054) alongside the root mean square error of approximation (RMSEA) indicating acceptable fit (0.069). The standardized root mean square residual (SRMR) indicated minimal discrepancy between observed and predicted correlations (0.054). The Tucker-Lewis Index stipulated moderate reliability (0.871^26^).

**Fig. 1:**
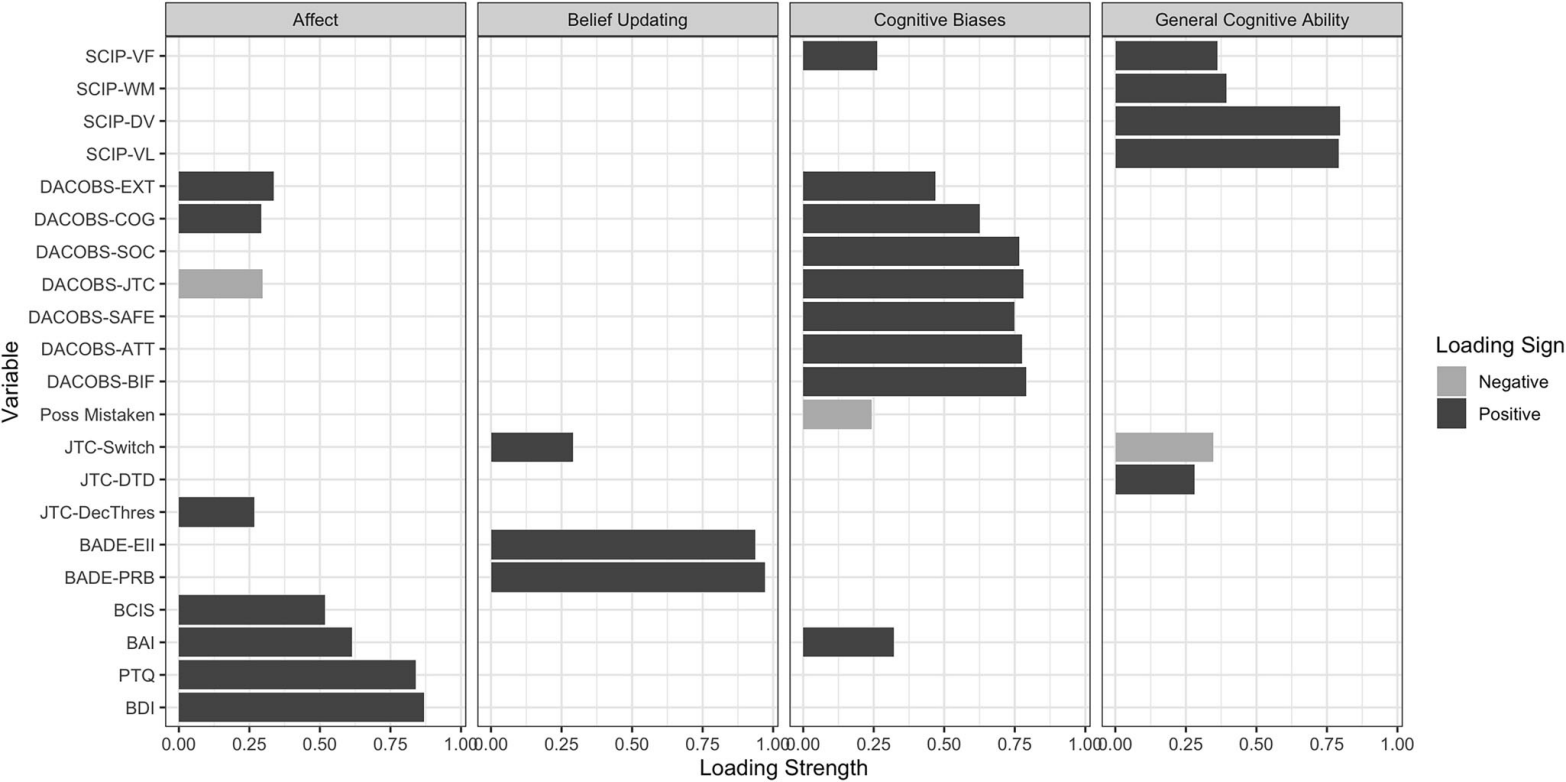
Factor loadings for 4 latent factors in SSD group (*N* = 75; left to right: (1) affect, (2) belief updating, (3) cognitive biases, and (4) general cognitive ability. Bar length indicates absolute loading strength and bar color indicates loading direction. Variable abbreviations in order of appearance: SCIP-VF verbal fluency from the screening for cognitive impairment in psychiatry, SCIP-WM working memory, SCIP-DV delayed verbal learning, SCIP-VL immediate verbal learning, DACOBS-EXT external attribution bias from the davos assessment of cognitive biases scale, DACOBS-COG subjective cognitive problems; DACOBS-SOC social cognition problems, DACOBS-JTC jumping to conclusions bias, DACOBS-SAFE safety behaviors, DACOBS-ATT attention for threat, DACOBS-BIF belief inflexibility, poss mistaken possibility of being mistaken, JTC-Switch switch number from jumping to conclusions task, JTC-DTD draws to decision, JTC-DecThres decision threshold, BADE-EII evidence integration impairment from bias against disconfirmatory evidence task, BADE-PRB positive response bias, BCIS Beck Cognitive Insight Scale, BAI Beck Anxiety Inventory, PTQ Perseverative Thinking Questionnaire, BDI Beck Depression Inventory.

Factor loadings are visualized in Fig. 1. A table of standardized loadings can be found in the Supplementary Table S1. Self-reports that capture components of emotional difficulties, such as the BDI, PTQ, and BAI, as well as the BCIS, predominantly comprise our Affect factor. The Belief Updating factor is driven by our two BADE metrics (evidence integration impairment, EII; positive response bias; PRB), which both capture some aspect of updating one’s beliefs based on new information. DACOBS subscores converge onto our Cognitive Biases factor, and SCIP subscores drive our fourth factor, labeled General Cognitive Ability. Importantly, JTC task metrics diverge onto different underlying factors. Draws to decision (DTD) aligns with General Cognitive Ability. Switching behavior (Switch) coincides with Belief Updating and inversely loads with General Cognitive Ability. Finally, decision threshold (DecThres) loads with Affect.

Both JTC’s Decision Threshold and the possibility of being mistaken had primary absolute loadings near the a priori cutoff of ±0.25 (DecThres = 0.267 with Affect; Poss Mistaken = −0.249 with Cognitive Biases). Sensitivity analyses were performed on model results with and without the inclusion of these variables. Neither significant explanatory variables nor model fit metrics changed, likely due to the variables’ weak loadings. Because of their relevance to our study, subsequent statistics are reported on models that include these variables.

#### Associations with clinical delusion severity

One individual did not report their sex, leaving 74 individuals for further analysis. For the PSYRATS total score as the dependent measure (Adj *R*^2^ = 0.368), Affect was the only significant predictor (corrected *p* < 0.001, *η*_*p*_^2^ = 0.27). Similarly, Affect significantly predicted almost all aspects of delusional ideation, aside from conviction (Preoccupation: corrected *p* = 0.04, *η*_*p*_^2^ = 0.12; Disruption: corrected *p* < 0.001, η_*p*_^2^ = 0.28; Distress: corrected *p* < 0.001, *η*_*p*_^2^ = 0.32). Distress was further explained by Belief Updating (uncorrected *p* = 0.016, *η*_*p*_^2^ = 0.14), and Conviction was explained only by Belief Updating (uncorrected *p* = 0.015, *η*_*p*_^2^ = 0.06). Of note, after Bonferroni correction, Belief Updating was marginally significant in our Conviction model (corrected *p* = 0.060) and Distress model (corrected *p* = 0.066), while all other relationships upheld after correction. See Table 3 for full model results.

**Table 3:** Multivariable linear regression outputs with PSYRATS (1) total, (2) preoccupation, (3) conviction, (4) distress, and (5) disruption as outcome.

PSYRATS	Total	Preoccupation	Conviction	Distress	Disruption
Cognitive biases	0.593 (0.672)	0.221 (0.354)	0.288+ (0.163)	−0.113 (0.301)	0.197+ (0.107)
Affect	2.832*** (0.668)	0.932** (0.351)	−0.008 (0.162)	1.443*** (0.300)	0.466*** (0.106)
Belief updating	−0.144 (0.635)	0.125 (0.334)	0.385* (0.154)	−0.701* (0.285)	0.047 (0.101)
General cognitive ability	0.863 (0.647)	0.434 (0.341)	0.026 (0.157)	0.305 (0.290)	0.098 (0.103)
N	74	74	74	74	74
*R*^2^	0.420	0.234	0.173	0.457	0.422
Adj *R*^2^	0.368	0.166	0.099	0.409	0.371
AIC	463.8	368.8	254.0	345.1	191.7
BIC	482.2	387.2	272.4	363.5	210.2
F	8.093	3.416	2.332	9.416	8.168
RMSE	4.99	2.62	1.21	2.24	0.79

Significance stars are assigned using uncorrected *p*-values, and standard errors for each Beta coefficient are in parentheses. Main effects of interest in **bold** survived Bonferroni correction at the *p* < 0.05 level.

+*p* < 0.1, *p < 0.05, **p < 0.01, ***p < 0.001.

#### Associations with unusual thought content

Table 4 illustrates estimations for five linear models in our SSD sample. Two individuals did not complete the survey and were removed from this analysis, yielding a total of 72 individuals. Seven individuals were further removed from PDI subscore models as they did not endorse any statements and as such, did not answer questions regarding preoccupation, conviction, or distress, for a total of 65 individuals. Inputted independent variables are identical to the above models.

**Table 4:** Multivariable linear regression outputs in SSD with PDI (1) endorsement (2) severity (= preoccupation + conviction + distress) (3) preoccupation (4) conviction (5) distress as outcome.

PDI	Total endorsement (yes/no)	Severity	Preoccupation	Conviction	Distress
Cognitive biases	2.019*** (0.503)	−0.098 (0.286)	0.025 (0.115)	−0.041 (0.138)	−0.083 (0.114)
Affect	1.834** (0.497)	1.537*** (0.283)	0.642*** (0.113)	0.143 (0.136)	0.752*** (0.112)
Belief updating	1.063* (0.480)	−0.065 (0.299)	0.030 (0.120)	0.086 (0.144)	−0.182 (0.119)
General cognitive ability	1.279* (0.484)	0.236 (0.276)	0.128 (0.111)	0.095 (0.133)	0.012 (0.110)
N	72	65	65	65	65
*R*^2^	0.441	0.389	0.393	0.058	0.533
Adj *R*^2^	0.389	0.326	0.331	-0.039	0.484
AIC	408.7	290.2	171.4	195.1	170.1
BIC	426.9	307.6	188.8	212.5	187.5
F	8.537	6.158	6.269	0.598	11.014
RMSE	3.70	1.99	0.80	0.96	0.79

Significance stars are assigned using uncorrected *p*-values and standard errors for each coefficient are in parentheses. Main effects of interest in **bold** survived Bonferroni correction at the *p* < 0.05 level.

*p < 0.05, **p < 0.01, ***p < 0.001.

After correction, the number of unusual beliefs endorsed was significantly associated with Affect (corrected *p* = 0.002, *η*_*p*_^2^ = 0.18), Cognitive Biases (corrected *p* < 0.001, *η*_*p*_^2^ = 0.25), and General Cognitive Ability (corrected *p* = 0.041, *η*_*p*_^2^ = 0.08). Belief Updating did not survive (corrected *p* = 0.122, *η*_*p*_^2^ = 0.07). Preoccupation (corrected *p* < 0.001, *η*_*p*_^2^ = 0.36) and Distress (corrected *p* < 0.001, *η*_*p*_^2^ = 0.49), but not Conviction (uncorrected *p* > 0.05, *η*_*p*_^2^ = 0.02), were also significantly associated with Affect.

## Discussion

Here, we present a comprehensive analysis of multiple cognitive and psychological processes hypothesized to contribute to delusion severity in participants with SSDs. Few studies to date have examined measures of cognition, affect, task-based reasoning biases, and self-reported cognitive biases simultaneously in a large cohort of individuals with delusional thought content. We first replicate prior work and observed greater task-based JTCs, BADE, and self-reported cognitive biases in SSD compared with non-clinical comparison (NCC) participants. We then find that task-based reasoning biases (Belief Updating) do capture unique variance beyond self-reported cognitive biases, and further that they are the only measures related to delusional conviction. We further extend research demonstrating a critical role of affective processes (e.g., mood, anxiety, perseveration) in delusional distress and preoccupation. Finally, we show no robust evidence of general cognitive ability explaining either delusion severity or reasoning and cognitive biases. We believe our approach can help disentangle overlapping variance between these features, enabling clearer mechanistic models of delusional severity that adequately account for multidimensional experiences.

### JTC divergence

To better understand the latent structure of delusions’ contributory factors, we conducted an exploratory factor analysis. We found evidence that cognitive ability, affect, and reasoning biases represent independent and unique factors. The one exception was in JTC, whose metrics diverged onto different latent factors. Draws to decision—the amount of information needed to make a conclusion—loaded onto General Cognitive Ability, suggesting that hasty decision-making on this task is related to poorer cognition, rather than a process akin to reasoning/cognitive biases. This finding is supported by previous work suggesting that reasoning biases may, in part, be a byproduct of cognitive deficits^17^ or even a result of carelessness and inattention^27^. However, more recent findings utilizing updated versions of the task—in similar fashion to ours—report the JTC bias is most likely a multicausal phenomenon capturing numerous underlying processes^28^. The present data support this nuanced independence from general cognitive ability.

JTC decision threshold—the probability assigned to the chosen lake at initial decision—was positively associated with scores on our Affect self-reports (i.e., BAI, BDI, PTQ). This suggests SSD participants with increased levels of anxiety, depression, or perseverative thinking tended to make decisions once they felt more confident. Mood disorders have uniquely been associated with requiring increased confidence for decision-making^29,30^, further suggesting that affective disturbances in SSD also contribute to seeking out more certainty before deciding.

Finally, JTC switch cross-loaded positively onto Belief Updating and negatively onto general cognitive ability. Considerable research has observed aspects of altered decision-making under uncertainty as a reflection of abnormal belief updating processes in paranoia^31,32^ as well as delusional psychosis more broadly^11,33,34^. Excessive switching, or frequently changing conclusions in the face of new information, may represent another manifestation of this distinct learning process. Altogether, divergence in aspects of JTC within the factor analysis suggests that the fishing task captures various dimensions of reasoning biases, and further that reasoning is not immune from other processes, such as affect. This aligns with prior work in clinical and non-clinical samples showing that valenced stimuli heightens susceptibility to logical fallacy (see ref. ^35^, for a review). While negative emotion is associated with more systematic reasoning^36^, it has not been shown to improve or “correct” reasoning, particularly when judgments may have been influenced by initial information. Our JTC findings support this unique discrepancy, in which decision threshold is high, yet SSD participants continue to make decisions with less information.

Future work would benefit from continuing to consider these higher-order processes in tandem—particularly in clinical samples where less ground has been covered yet preliminary work such as this supports their mutual impact. Studies with larger sample sizes, and thus more statistical power, are also needed to determine whether this particular divergence is stable, as loadings—notably for JTC’s Decision Threshold—were low.

### Affect and delusions

In line with prior studies, Affect factor scores upheld robust, significant relationships with delusional distress^37^ and preoccupation^38^. This was seen in both clinical delusions (PSYRATS) and delusional ideations (PDI), suggesting that affective disturbances relate to distress associated with specific delusional beliefs as well as broader instances of unusual thought content. There is a wealth of literature supporting the substantial role affect plays in delusion severity, particularly distress^39–41^. Our models suggest that reducing negative affect could improve delusional distress, or vice versa.

### Neurocognition and delusions

In this sample, general cognitive ability did not show robust associations with delusional severity when affective and reasoning-related factors were also considered. The only aspect of delusional thinking that related to neurocognitive ability in our sample was the number of delusional ideation statements endorsed on the PDI. The lack of a strong association between delusional severity and cognitive deficits is elusive, given the pervasive co-occurrence of the two phenotypes in psychotic disorders^16,25^. There have been several attempts to determine the relationship between basic neurocognitive function and delusions, with recent large-sample work showing only modest, albeit significant associations^42^. While speculative, the relationship with item endorsement on the PDI suggests that vulnerability to delusions (i.e., willingness to endorse unusual or unlikely beliefs) relates to neurocognition, but severity is more strongly related to higher-order cognition (i.e., reasoning biases) and affect. It should be noted, however, that the IQ restrictions on our study’s inclusion criteria and the timing of assessments being shortly after hospitalization, may have influenced these results. Future studies unbound by these restrictions are needed to bolster this finding.

### Biases and delusions

Cognitive Biases—driven by responses on the DACOBS self-report— were significantly related to PDI’s total endorsement. This aligns with prior findings that cognitive biases are broadly linked to a vulnerability to delusions^43^. As the DACOBS captures one’s self-reported propensity toward various cognitive distortions or biases (e.g., belief inflexibility, low data-gathering), it is possible subjective and objective assessments have differential contributions, an argument supported by unique clinical correlates seen in task-based versus self-reported JTC bias^44^.

Notably, the only factor showing an association with delusional conviction was Belief Updating, which included poorer evidence integration and greater PRB on the BADE, as well as excessive switching during JTC. These learning-based processes reflect how individuals integrate new information into their beliefs. Belief updating itself is multifaceted, requiring optimal weighting of new information, a willingness to update priors based on incoming data, and an accurate representation of uncertainties within the environment^45^. Our data suggests that greater difficulties with this process are linked with more strongly endorsing a delusional belief, particularly the clinical delusion that led them to seek higher levels of psychiatric care. This specificity to clinical delusional severity is further supported by the lack of relationship between belief updating and broader delusional ideation (PDI). Notably, this is in line with a recent meta-analysis that did not find a link between JTC and PDI^46^. Psychological treatments targeting the counterweighting of beliefs and the weighing of new information may be particularly helpful in reducing conviction of clinical delusions^3,47^. Also interesting was that, despite relevance to every other aspect of severity, Affect factor scores were not tied to conviction in either delusion measure, highlighting a possible specificity to belief updating that is important to continue researching.

### Limitations and future directions

Despite several strengths, our analysis also had limitations. Firstly, a factor loading cutoff (>0.25) was used to balance between validity and interpretability of results. This score is generally considered low, but as the goal of this analysis was to capture a complex interplay of cognitive, affective, and reasoning structures, we did not employ a more stringent cutoff. Likewise, we did not wish to be too lenient, as allowing for many weak loadings reduces interpretability and increases noise. We did attempt to strengthen our EFA by calculating an MSA prior to analyses as a validated way of debugging unsuitable or noisy variables^48^. Further, the decision to use mean imputation was made at the possible sacrifice of lost variance. However, the low proportion of missing data (<1%) was below level of concern for this. Further, multiple imputation was alternatively tested and yielded comparable results. We believe that this serves as a strength to our models and may mitigate concerns about the use of mean imputation.

Prior studies utilizing this version of the JTC fishing task have noted a vulnerability to comprehension/attention (e.g., ref. ^28^). While our study is not immune to these same concerns, we hope to have lowered this risk by having participants complete these tasks with a staff member present. Staff were instructed to read all instructions aloud, answer any comprehension questions, and observe the participant as they were completing the task to ensure they were paying attention.

Future studies utilizing EFA would benefit from obtaining larger sample sizes. Our sample size is considered adequate by some standards^49^ but below the minimum recommended for a four-factor solution by others^50^. As such, a larger sample size is needed to determine how robust these factors are. Other variables, such as antipsychotic medication, must also be considered due to their subjective effects on cognitive and affective experiences^51^. Further, the factor solution might look different in samples with more severe cognitive impairment, as mentioned above. As such, associations should be appraised within the bounds of our sample’s demographic makeup. Future researchers should continue to disentangle the relationship between measures of reasoning biases—particularly belief updating—and delusional severity. Longitudinal designs would represent a next step for helping us to better understand whether these measures move in tandem with delusion severity, and thus provide more support of their interventional utility.

## Conclusion

The present study examined the latent structure of both task-based and self-reported reasoning biases, alongside cognition and affect, to determine how factors relate to facets of delusional severity. Results illustrated that reasoning biases do uniquely explain variance in SSD above and beyond cognitive deficits, and further that measures of belief updating are directly associated with conviction of a core clinical delusion and endorsement of broader unusual thought content. They further demonstrate the critical role of affect in all facets of delusion severity, except conviction, suggesting specific associations between both belief updating and conviction and affect and preoccupation/distress. Findings suggest that reasoning biases—particularly those related to belief updating—may be a vital contributor to the strength with which maladaptive beliefs are held, necessitating continued exploration of diverse, reachable mechanisms of the delusional experience.

## Methods

Individuals with a primary psychotic disorder and active delusion, aged 18-55, were identified through Vanderbilt Psychiatric Hospital’s inpatient and partial hospitalization programs (PHP) for a study focused on recovery from an acute psychotic episode, recruiting from 2020 to 2025. All participants had been discharged from the inpatient unit (*n* = 70) or PHP (*n* = 5) within 3.5 months of completing assessments (average = 32.19 days, range = −2–104 days). Seventy-five participants were enrolled (35 schizophrenia, 20 schizoaffective, 13 schizophreniform, three brief psychotic disorder, one delusional disorder, and three psychosis not otherwise specified). Diagnosis was determined based on the inpatient discharge diagnosis given by the attending physician (*N* = 73) or using the Structured Clinical Interview of the DSM-5 (SCID) by a trained clinician, if available (*N* = 2). Exclusion criteria included major physical or neurological illness, active substance use disorder, history of traumatic brain injury, and an estimated IQ < 70. Seventy NCC participants were additionally recruited via existing study registries at Vanderbilt and community advertisement. NCC participants were all assessed via SCID to ensure they did not meet criteria for any current psychiatric disorder and were not being prescribed psychotropic medication; however, they were included if they screened positive for a past (>6 months ago) mild depressive episode or anxiety disorder, given the symptoms were resolved and the participant was not receiving psychiatric treatment. NCC participants further could not have a first-degree relative with a diagnosed psychotic disorder. SSD and NCC groups were matched on age, sex, and parental education.

The study was approved by Vanderbilt University Medical Center’s Institutional Review Board (IRB). The authors assert that all procedures contributing to this work comply with the ethical standards of the relevant national and institutional committees on human experimentation and with the Helsinki Declaration of 1975, as revised in 2008. All participants provided written informed consent prior to participating in any study activities.

Participants were given the option of completing the study in-person (76.6%) or virtually (23.4%). These data were collected as part of a longitudinal study; here we report only data from the initial assessment. This study was not preregistered. De-identified, raw data may be accessed through a link provided in the Supplement.

### Delusional severity

#### Psychotic symptom rating scale for delusions (PSYRATS-Delusions)

Participants created a summary statement meant to capture the core of their delusional belief (e.g., “my neighbor is monitoring me through cameras in my home”) and were then asked six questions about their preoccupation (amount/intensity), distress (amount/intensity), conviction, and functional disruption related to that belief, with each item rated from 0 (absent) to 4 (severe^52^). The PSYRATS provides a multidimensional assessment of delusions and has demonstrated good reliability^53^.

#### Peters delusion inventory (PDI-21)

The 21-item version of the Peters Delusion Inventory^23^ captures broad unusual thought content. Beyond binary endorsement (yes/no) of 21 delusional statements (e.g., “Do you ever feel as if things in magazines or on TV were written especially for you?”), the survey uses a 5-point Likert scale to capture dimensions of distress, preoccupation, and conviction. Here, we sum each metric across all endorsed beliefs and then divide by the number of endorsed beliefs, to obtain a dimension score that is independent from number of items endorsed. The PDI has maintained invariance across time and samples^54^.

### Reasoning biases

#### Bias against disconfirmatory evidence (BADE)

The scenario task designed by Woodward, Moritz, Cuttler, and Whitman^55^ was used to measure one’s BADE. Participants completed examples with staff present, who verbally explained the task alongside written instructions. Staff also remained present to answer any questions throughout the task and ensure participants were attending to the rules. The abbreviated paradigm includes six scenarios and four explanations for each scenario. Three facts are provided iteratively, and participants are asked to update their beliefs about the plausibility of various explanations. Of the four explanations, one is “Absurd” (consistently improbable), one is “True” (consistently probable and considered most likely by the end), and two are “Lures” (initially plausible but decreasingly so as new information is presented).

Two metrics were calculated from the BADE^56^. EII captures one’s inability to incorporate new information into preexisting beliefs, equal to the sum of averaged Absurd scores across all trials and the averaged Lure scores from the third exposure (i.e., final fact) only. PRB captures one’s liberal response style and is calculated as the sum of Lure and True scores.

#### Jumping to conclusions (JTC)

JTC bias can be measured in a task-based fashion with the well-validated fishing game^57^. Across ten rounds, participants are asked to rate the likelihood that a fisherman is fishing from one of two lakes, given inverted proportions of two differently colored fish in each lake (80:20 and 20:80). Each round, participants rate how probable it is the fisherman is fishing from each lake and can further pick a lake or say they do not have enough information to decide. Similarly to the BADE task, staff were present throughout the duration of the task to answer questions and ensure attention.

Data was scored into three metrics. Draws to decision (DTD) reflects how many trials (draws from the lake) it took for the participant to make an initial decision about which lake was being fished from, with lower numbers indicating greater JTC bias^58^. Decision threshold (DecThres) represents the probability players give to their lake being the correct lake after the initial decision is made, with lower values reflecting a propensity for low-confidence decision-making^59^. Switching behavior (Switch) is the number of times the participant changed their decision about which lake was being fished from throughout the ten rounds. Excessive switching may reflect unstable estimations of the task environment^33^.

#### Possibility of being mistaken

The possibility of being mistaken is one measure designed to capture belief flexibility^60^. In relation to SSD PSYRATS statements, participants are asked “on a scale of 0%–100%, how likely is it that you are mistaken about this belief?” Higher scores indicate greater belief flexibility.

#### Davos assessment of cognitive biases scale (DACOBS)

The Davos Assessment of Cognitive Biases Scale is a reliable and validated self-report developed to assess an array of cognitive biases relevant for maintaining psychotic experiences: JTC, social cognition, subjective cognitive problems, attention for threat, external attribution, belief inflexibility, and safety behaviors^61^. Participants are asked how strongly they agree or disagree with 42 statements using a 7-point Likert scale.

#### Beck cognitive insight scale (BCIS)

Cognitive insight was captured with the Beck Cognitive Insight Scale, a 14-item self-report^15^. Nine items measure self-reflectiveness and five items measure self-certainty. The composite score is calculated by subtracting self-certainty subtotals from self-reflectiveness subtotals, with higher scores indicating better cognitive insight. The BCIS has been shown to track changes in delusional beliefs and predict treatment outcomes^62^.

### Affect

#### Beck depression inventory (BDI-II)

Depressive symptoms were captured with the Beck Depression Inventory II, a 21-item self-report^63^. The BDI-II is well-validated, widely used, and has been replicated across numerous populations.

#### Beck anxiety inventory (BAI)

The Beck Anxiety Inventory is a 21-item self-report that measures severity of anxiety symptoms^64^.

#### Perseverative thinking questionnaire (PTQ)

The Perseverative Thinking Questionnaire consists of 22 items describing one’s typical response to sad moods or negative experiences^65^. Individuals are asked to rate on a 5-point Likert scale how often they endorse experiences of repetitive negative thinking, from 0 (never) to 4 (always).

### Cognition

#### Screen for cognitive impairment in psychiatry (SCIP)

Cognitive ability was measured with the SCIP^66^, which included measures of immediate and delayed verbal learning, verbal fluency, working memory, and visuomotor learning. Scores were standardized based on published norms using Z-scores in analyses^67^. The SCIP is a validated and reliable measure of cognitive ability in both psychiatric and non-psychiatric populations.

### Statistical analysis

All analyses were performed in RStudio (v.2025.05.1 + 513). To validate assumptions of general impairment in our SSD group, we began by assessing group differences in key variables of interest, including affective disturbances, task-based reasoning biases, and self-reported cognitive biases. We conducted a series of Type-III Analyses of Covariance (ANCOVA) models using the *car* package^68^, with group (SSD/NCC) as the between-subjects factor. We then used adjusted means to calculate Tukey’s Honest Significant Difference, post-hoc^69^.

To investigate potential latent factors in our SSD group, an exploratory factor analysis (EFA) was performed with *psych*^70^ and *GPArotation*^71^ packages. Included measures are: verbal fluency, verbal learning, delayed verbal learning, and working memory from the SCIP; BCIS total; BDI total; BAI total; EII and PRB from BADE; draws to decision, decision threshold, and number of switches from JTC; possibility of being mistaken; and all seven DACOBS subscales. To determine adequacy of our sample for this approach, we first performed Bartlett’s test of Sphericity^72^ and Kayser-Meyer-Olkin’s (KMO) test^73^. Measures were further evaluated with the Measure of Sampling Adequacy (MSA) prior to running the EFA^74^. A parallel analysis using eigenvalues was then performed to determine the optimal number of factors^75^. We applied an oblimin (oblique) rotation as we expected underlying factors—particularly amongst variables under the same assessment—to be correlated with each other^76^. Principal axis factoring was assigned as the extraction method. The tenBerge was chosen as the method for estimating factor scores, as it is considered a robust alternative to regression^77^. We chose to use a factor loading cutoff of >0.25 to balance interpretability and inclusion of conceptually relevant variables. We further performed various sensitivity analyses with alternative specifications to ensure main results were unchanged.

To examine unique and meaningful contributions of factors on delusional dimensions in our SSD group only, multivariable linear regression models were created using *lme4*^78^ and *lmerTest*^79^ packages. Models were constructed to predict each dimension of delusional thought content with main effects of age, sex, and factor scores. Prior to analysis, standard assumptions were met. Standardized Beta coefficients (*β*) and associated *p*-values are reported to illustrate the strength and significance of each predictor. *R*^2^, Adjusted *R*^2^, F-statistic, and Root Mean Square Error (RMSE) are reported to cohesively assess each model’s overall goodness-of-fit to the data. Both uncorrected and Bonferroni-corrected *p*-values are reported. Bonferroni corrections are used as a conservative approach, though uncorrected *p*-values and effect sizes are also considered when interpreting exploratory findings, particularly where theoretical relevance is high.

Mean imputation was used for missing variables for simplicity and to preserve sample size, ensuring SSD and NCC groups were performed separately. While this method may underestimate variance, it was considered appropriate given the low proportion of missing data (0.64%^80^). We plotted histograms before and after imputation to ensure distributions were visually unchanged.

## Supplementary information

Relating latent factors of reasoning, affect, and cognition to the delusional experience: Supplement

## Data Availability

De-identified, raw data used for present analyses can be accessed through: https://github.com/annalisehalverson/CogAffReasonDelFA.git. This article has been published as a preprint on PsyArXiv: 10.31234/osf.io/k95wj_v1.
