# Supplementary material for: Relating latent factors of reasoning, affect, and cognition to the delusional experience
Source: Schizophrenia (Heidelb). 2026 Apr 24;12(1):57. doi: 10.1038/s41537-026-00750-1 (PMC13319119; doi:10.1038/s41537-026-00750-1)
Supplement: Supplementary file 1 — Relating latent factors of reasoning, affect, and cognition to the delusional experience: Supplement [file 41537_2026_750_MOESM1_ESM.docx]

Relating latent factors of reasoning, affect, and cognition to the delusional experience: Supplement

***Figure S1: Parallel Analysis Scree Plot for EFA***

The dark grey line represents observed eigenvalues; the light grey line represents random eigenvalues. Retained factors have observed eigenvalues that are greater than random eigenvalues. The point where observed eigenvalues fall below random eigenvalues (illustrated here with a vertical black line) indicates the cutoff of number of factors to keep; here, a four-factor structure was revealed.


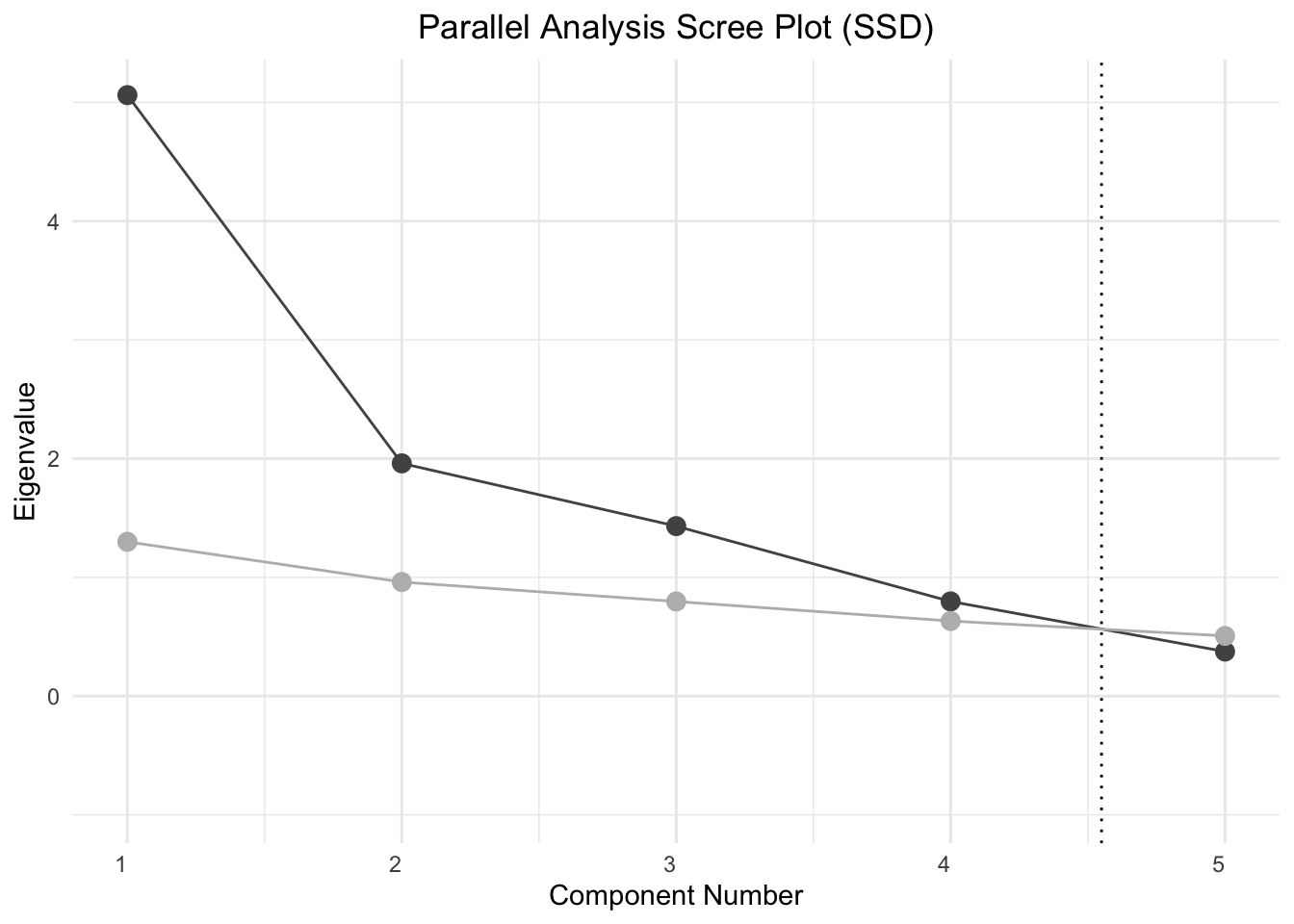


***Table S1: Four-factor model standardized loadings of exploratory factor analysis***

Values in bold represent the highest absolute loading for each variable.

| **Variable** | **Cognitive Biases** | **Affect** | **Belief Updating** | **General Cognitive Ability** |
| --- | --- | --- | --- | --- |
| SCIP-VF | 0.263 | -0.078 | 0.194 | **0.362** |
| SCIP-WM | -0.018 | -0.023 | -0.093 | **0.393** |
| SCIP-DV | -0.051 | 0.040 | -0.062 | **0.796** |
| SCIP-VL | -0.060 | -0.017 | -0.053 | **0.791** |
| DACOBS-EXT | **0.468** | 0.336 | -0.064 | -0.237 |
| DACOBS-COG | **0.627** | 0.292 | -0.085 | -0.037 |
| DACOBS-SOC | **0.765** | 0.231 | -0.044 | -0.031 |
| DACOBS-JTC | **0.780** | -0.297 | 0.143 | 0.036 |
| DACOBS-SAFE | **0.748** | 0.034 | -0.050 | -0.094 |
| DACOBS-ATT | **0.775** | 0.158 | -0.029 | 0.009 |
| DACOBS-BIF | **0.790** | -0.197 | -0.113 | -0.094 |
| Poss Mistaken | **-0.242** | 0.104 | -0.056 | 0.006 |
| JTC-Switch | 0.041 | 0.109 | 0.290 | **-0.348** |
| JTC-DTD | -0.238 | 0.061 | -0.004 | **0.281** |
| JTC-DecThres | 0.101 | **0.267** | -0.068 | 0.019 |
| BADE-EII | -0.021 | -0.029 | **0.936** | -0.016 |
| BADE-PRB | -0.035 | 0.035 | **0.970** | -0.046 |
| BCIS | 0.010 | **0.518** | 0.034 | 0.198 |
| BAI | 0.321 | **0.613** | 0.217 | 0.240 |
| PTQ | -0.014 | **0.840** | -0.029 | -0.119 |
| BDI | -0.020 | **0.870** | -0.017 | 0.021 |
